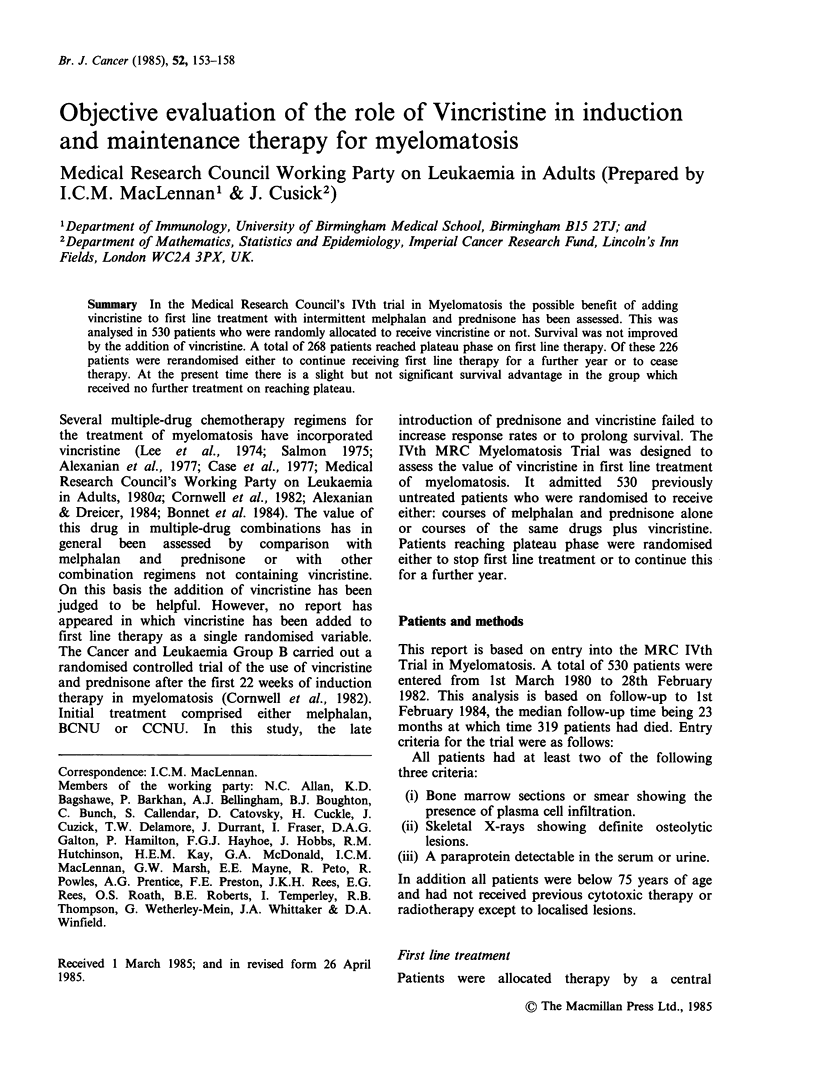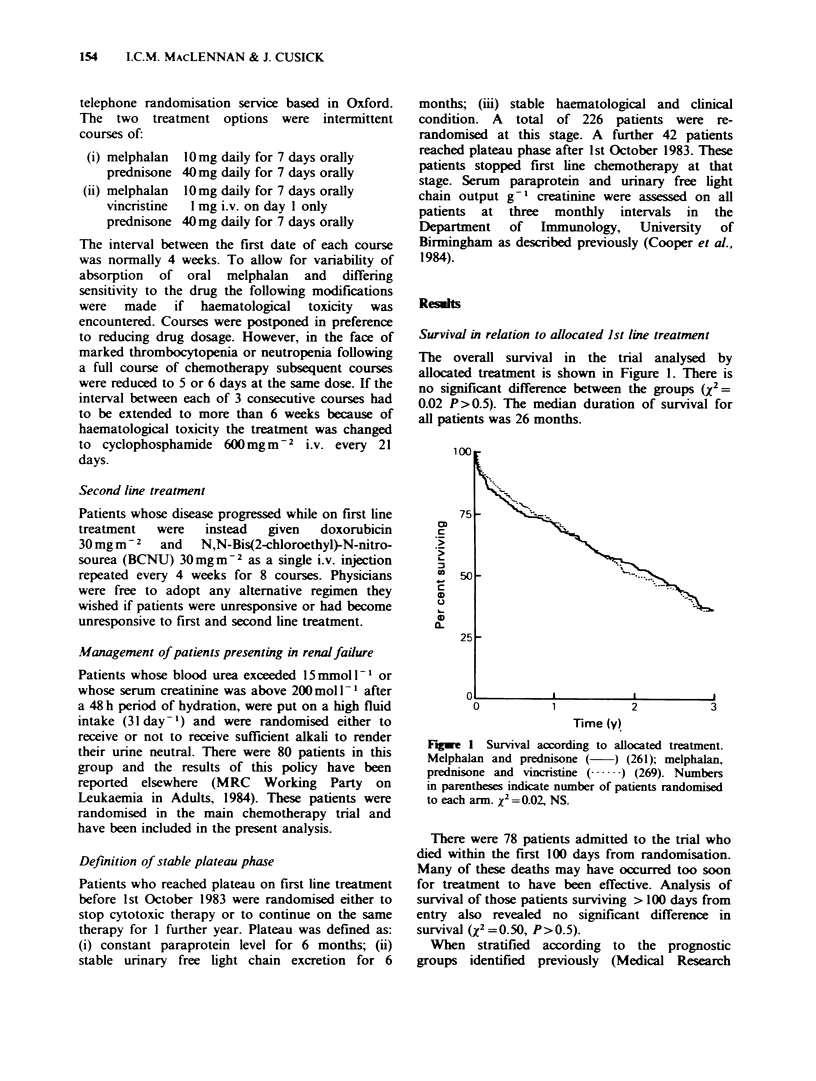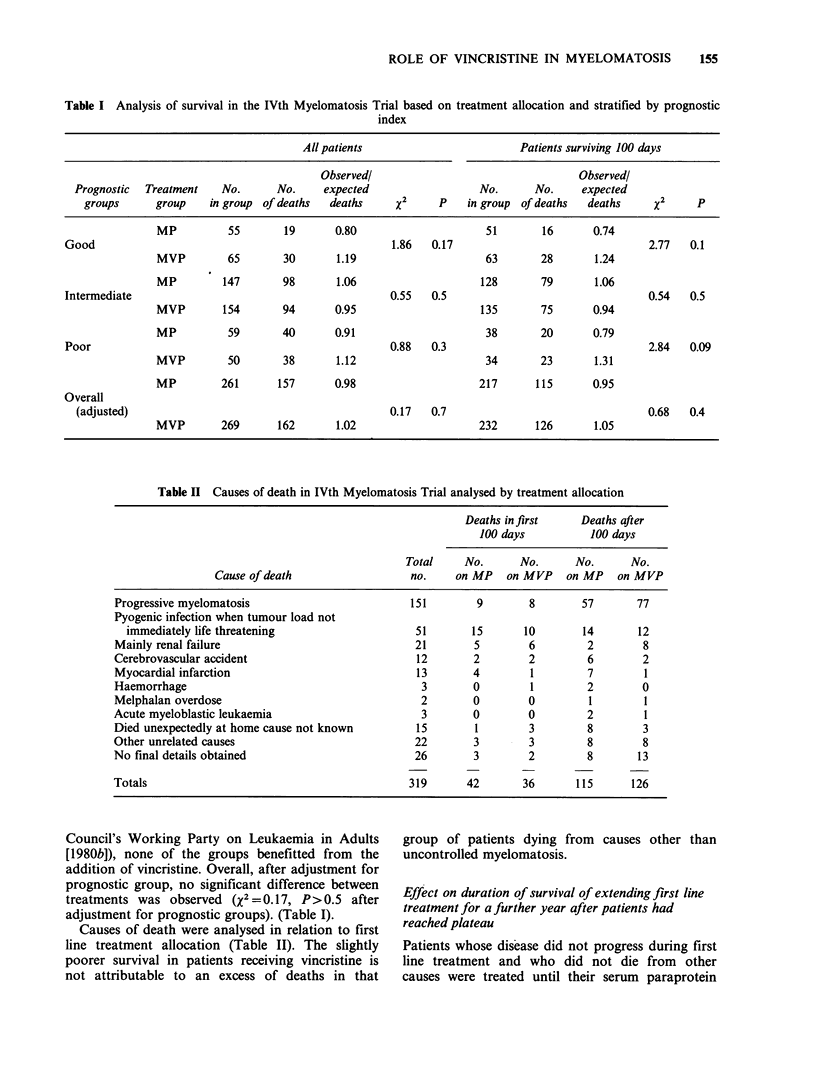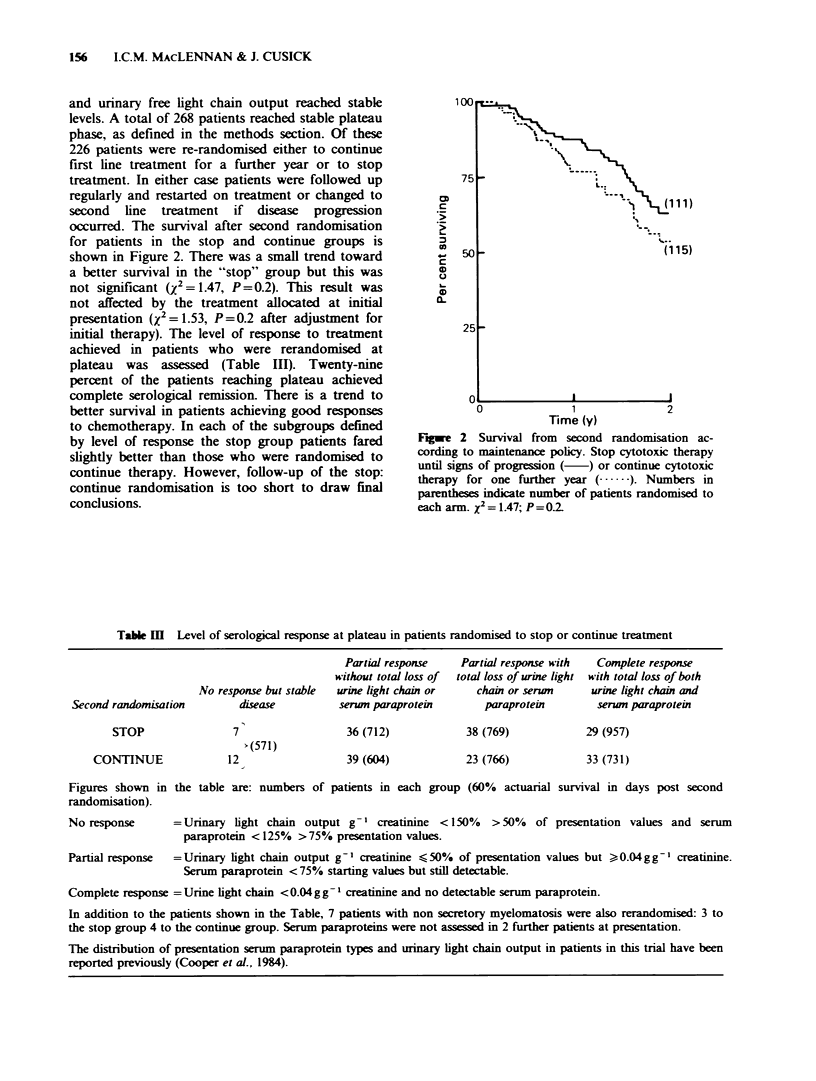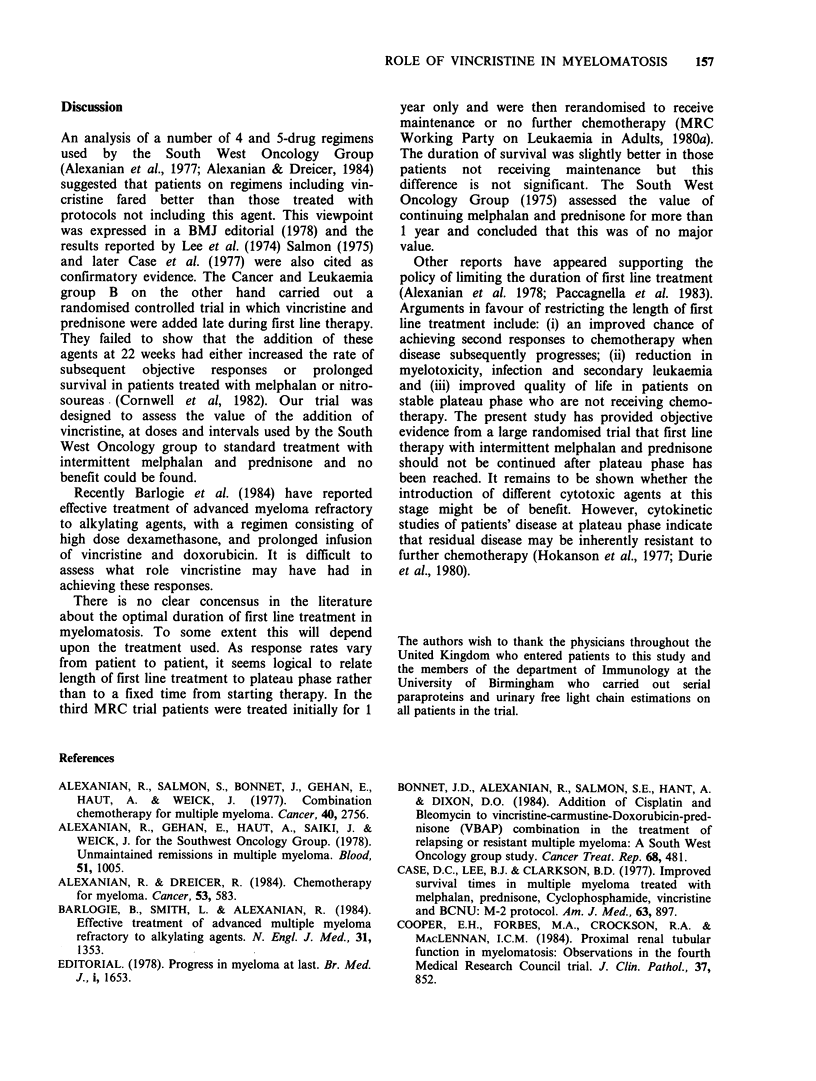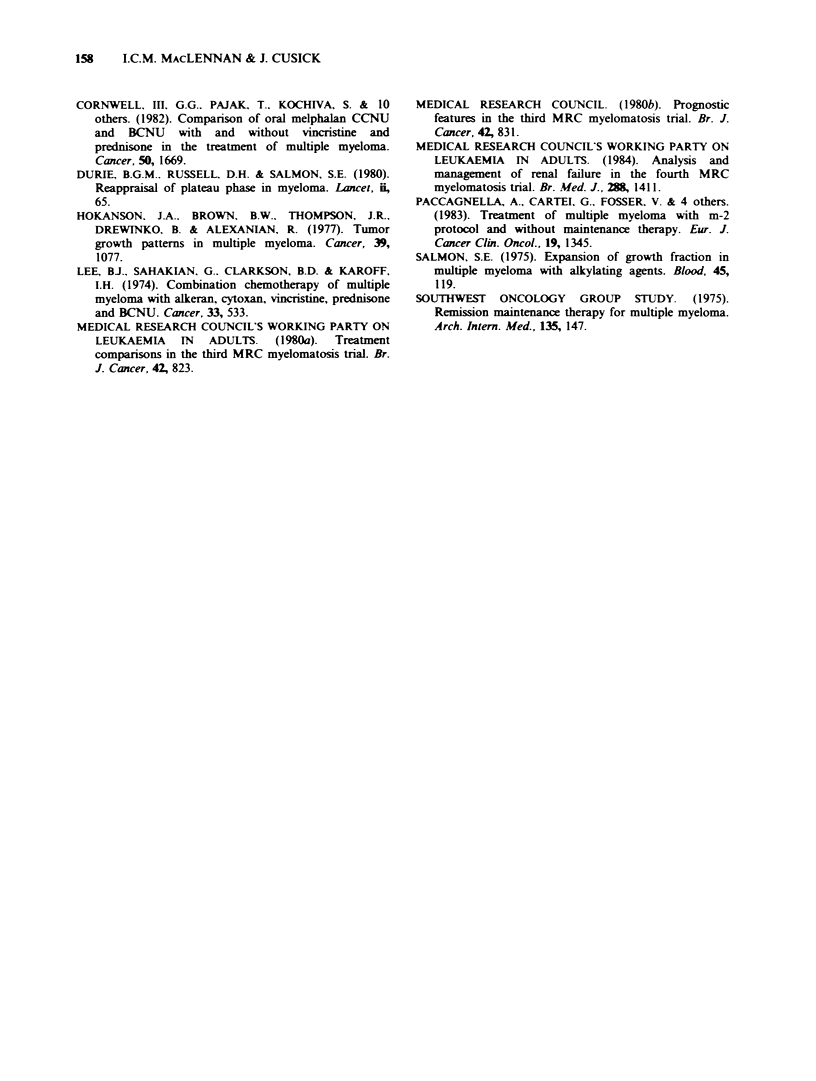# Objective evaluation of the role of vincristine in induction and maintenance therapy for myelomatosis. Medical Research Council Working Party on Leukaemia in Adults.

**DOI:** 10.1038/bjc.1985.171

**Published:** 1985-08

**Authors:** I. C. MacLennan, J. Cusick

## Abstract

In the Medical Research Council's IVth trial in Myelomatosis the possible benefit of adding vincristine to first line treatment with intermittent melphalan and prednisone has been assessed. This was analysed in 530 patients who were randomly allocated to receive vincristine or not. Survival was not improved by the addition of vincristine. A total of 268 patients reached plateau phase on first line therapy. Of these 226 patients were rerandomised either to continue receiving first line therapy for a further year or to cease therapy. At the present time there is a slight but not significant survival advantage in the group which received no further treatment on reaching plateau.


*Br. J. Cancer* (1985), **52**, 153–158

### Objective evaluation of the role of Vincristine in induction and maintenance therapy for myelomatosis

Medical Research Council Working Party on Leukaemia in Adults (Prepared by I.C.M. MacLennan[1] & J. Cusick[2])

[1]*Department of Immunology, University of Birmingham Medical School, Birmingham B15 2TJ; and* [2]*Department of Mathematics, Statistics and Epidemiology, Imperial Cancer Research Fund, Lincoln's Inn Fields, London WC2A 3PX, UK.*

**Summary** In the Medical Research Council's IVth trial in Myelomatosis the possible benefit of adding vincristine to first line treatment with intermittent melphalan and prednisone has been assessed. This was analysed in 530 patients who were randomly allocated to receive vincristine or not. Survival was not improved by the addition of vincristine. A total of 268 patients reached plateau phase on first line therapy. Of these 226 patients were rerandomised either to continue receiving first line therapy for a further year or to cease therapy. At the present time there is a slight but not significant survival advantage in the group which received no further treatment on reaching plateau.

Several multiple-drug chemotherapy regimens for the treatment of myelomatosis have incorporated vincristine (Lee *et al.*, 1974; Salmon 1975; Alexanian *et al.*, 1977; Case *et al.*, 1977; Medical Research Council's Working Party on Leukaemia in Adults, 1980*a*; Cornwell *et al.*, 1982; Alexanian & Dreicer, 1984; Bonnet *et al.* 1984). The value of this drug in multiple-drug combinations has in general been assessed by comparison with melphalan and prednisone or with other combination regimens not containing vincristine. On this basis the addition of vincristine has been judged to be helpful. However, no report has appeared in which vincristine has been added to first line therapy as a single randomised variable. The Cancer and Leukaemia Group B carried out a randomised controlled trial of the use of vincristine and prednisone after the first 22 weeks of induction therapy in myelomatosis (Cornwell *et al.*, 1982). Initial treatment comprised either melphalan, BCNU or CCNU. In this study, the late introduction of prednisone and vincristine failed to increase response rates or to prolong survival. The IVth MRC Myelomatosis Trial was designed to assess the value of vincristine in first line treatment of myelomatosis. It admitted 530 previously untreated patients who were randomised to receive either: courses of melphalan and prednisone alone or courses of the same drugs plus vincristine. Patients reaching plateau phase were randomised either to stop first line treatment or to continue this for a further year.

### Patients and methods

This report is based on entry into the MRC IVth Trial in Myelomatosis. A total of 530 patients were entered from 1st March 1980 to 28th February 1982. This analysis is based on follow-up to 1st February 1984, the median follow-up time being 23 months at which time 319 patients had died. Entry criteria for the trial were as follows:

All patients had at least two of the following three criteria:

(i) Bone marrow sections or smear showing the presence of plasma cell infiltration.
(ii) Skeletal X-rays showing definite osteolytic lesions.
(iii) A paraprotein detectable in the serum or urine.

In addition all patients were below 75 years of age and had not received previous cytotoxic therapy or radiotherapy except to localised lesions.

### *First line treatment*

Patients were allocated therapy by a central

Correspondence: I.C.M. MacLennan.

Members of the working party: N.C. Allan, K.D. Bagshawe, P. Barkhan, A.J. Bellingham, B.J. Boughton, C. Bunch, S. Callendar, D. Catovsky, H. Cuckle, J. Cuzick, T.W. Delamore, J. Durrant, I. Fraser, D.A.G. Galton, P. Hamilton, F.G.J. Hayhoe, J. Hobbs, R.M. Hutchinson, H.E.M. Kay, G.A. McDonald, I.C.M. MacLennan, G.W. Marsh, E.E. Mayne, R. Peto, R. Powles, A.G. Prentice, F.E. Preston, J.K.H. Rees, E.G. Rees, O.S. Roath, B.E. Roberts, I. Temperley, R.B. Thompson, G. Wetherley-Mein, J.A. Whittaker & D.A. Winfield.

Received 1 March 1985; and in revised form 26 April 1985.

© The Macmillan Press Ltd., 1985

telephone randomisation service based in Oxford. The two treatment options were intermittent courses of:

(i) melphalan 10 mg daily for 7 days orally
prednisone 40 mg daily for 7 days orally
(ii) melphalan 10 mg daily for 7 days orally
vincristine 1 mg i.v. on day 1 only
prednisone 40 mg daily for 7 days orally

The interval between the first date of each course was normally 4 weeks. To allow for variability of absorption of oral melphalan and differing sensitivity to the drug the following modifications were made if haematological toxicity was encountered. Courses were postponed in preference to reducing drug dosage. However, in the face of marked thrombocytopenia or neutropenia following a full course of chemotherapy subsequent courses were reduced to 5 or 6 days at the same dose. If the interval between each of 3 consecutive courses had to be extended to more than 6 weeks because of haematological toxicity the treatment was changed to cyclophosphamide 600 mg m$^{-2}$ i.v. every 21 days.

*Second line treatment*

Patients whose disease progressed while on first line treatment were instead given doxorubicin 30 mg m$^{-2}$ and N,N-Bis(2-chloroethyl)-N-nitrosourea (BCNU) 30 mg m$^{-2}$ as a single i.v. injection repeated every 4 weeks for 8 courses. Physicians were free to adopt any alternative regimen they wished if patients were unresponsive or had become unresponsive to first and second line treatment.

*Management of patients presenting in renal failure*

Patients whose blood urea exceeded 15 mmol l$^{-1}$ or whose serum creatinine was above 200 mol l$^{-1}$ after a 48 h period of hydration, were put on a high fluid intake (3 l day$^{-1}$) and were randomised either to receive or not to receive sufficient alkali to render their urine neutral. There were 80 patients in this group and the results of this policy have been reported elsewhere (MRC Working Party on Leukaemia in Adults, 1984). These patients were randomised in the main chemotherapy trial and have been included in the present analysis.

*Definition of stable plateau phase*

Patients who reached plateau on first line treatment before 1st October 1983 were randomised either to stop cytotoxic therapy or to continue on the same therapy for 1 further year. Plateau was defined as: (i) constant paraprotein level for 6 months; (ii) stable urinary free light chain excretion for 6 months; (iii) stable haematological and clinical condition. A total of 226 patients were re-randomised at this stage. A further 42 patients reached plateau phase after 1st October 1983. These patients stopped first line chemotherapy at that stage. Serum paraprotein and urinary free light chain output g$^{-1}$ creatinine were assessed on all patients at three monthly intervals in the Department of Immunology, University of Birmingham as described previously (Cooper *et al.*, 1984).

### Results

*Survival in relation to allocated 1st line treatment*

The overall survival in the trial analysed by allocated treatment is shown in Figure 1. There is no significant difference between the groups ($\chi^2 = 0.02$ $P > 0.5$). The median duration of survival for all patients was 26 months.

**Figure 1** Survival according to allocated treatment. Melphalan and prednisone (——) (261); melphalan, prednisone and vincristine (······) (269). Numbers in parentheses indicate number of patients randomised to each arm. $\chi^2 = 0.02$, NS.

There were 78 patients admitted to the trial who died within the first 100 days from randomisation. Many of these deaths may have occurred too soon for treatment to have been effective. Analysis of survival of those patients surviving >100 days from entry also revealed no significant difference in survival ($\chi^2 = 0.50$, $P > 0.5$).

When stratified according to the prognostic groups identified previously (Medical Research

**Table I** Analysis of survival in the IVth Myelomatosis Trial based on treatment allocation and stratified by prognostic index

| | | *All patients* | | | | | *Patients surviving 100 days* | | | | |
|---|---|---|---|---|---|---|---|---|---|---|---|
| *Prognostic groups* | *Treatment group* | *No. in group* | *No. of deaths* | *Observed/ expected deaths* | $\chi^2$ | *P* | *No. in group* | *No. of deaths* | *Observed/ expected deaths* | $\chi^2$ | *P* |
| Good | MP | 55 | 19 | 0.80 | 1.86 | 0.17 | 51 | 16 | 0.74 | 2.77 | 0.1 |
| | MVP | 65 | 30 | 1.19 | | | 63 | 28 | 1.24 | | |
| Intermediate | MP | 147 | 98 | 1.06 | 0.55 | 0.5 | 128 | 79 | 1.06 | 0.54 | 0.5 |
| | MVP | 154 | 94 | 0.95 | | | 135 | 75 | 0.94 | | |
| Poor | MP | 59 | 40 | 0.91 | 0.88 | 0.3 | 38 | 20 | 0.79 | 2.84 | 0.09 |
| | MVP | 50 | 38 | 1.12 | | | 34 | 23 | 1.31 | | |
| Overall (adjusted) | MP | 261 | 157 | 0.98 | 0.17 | 0.7 | 217 | 115 | 0.95 | 0.68 | 0.4 |
| | MVP | 269 | 162 | 1.02 | | | 232 | 126 | 1.05 | | |

**Table II** Causes of death in IVth Myelomatosis Trial analysed by treatment allocation

| | | *Deaths in first 100 days* | | *Deaths after 100 days* | |
|---|---|---|---|---|---|
| *Cause of death* | *Total no.* | *No. on MP* | *No. on MVP* | *No. on MP* | *No. on MVP* |
| Progressive myelomatosis | 151 | 9 | 8 | 57 | 77 |
| Pyogenic infection when tumour load not immediately life threatening | 51 | 15 | 10 | 14 | 12 |
| Mainly renal failure | 21 | 5 | 6 | 2 | 8 |
| Cerebrovascular accident | 12 | 2 | 2 | 6 | 2 |
| Myocardial infarction | 13 | 4 | 1 | 7 | 1 |
| Haemorrhage | 3 | 0 | 1 | 2 | 0 |
| Melphalan overdose | 2 | 0 | 0 | 1 | 1 |
| Acute myeloblastic leukaemia | 3 | 0 | 0 | 2 | 1 |
| Died unexpectedly at home cause not known | 15 | 1 | 3 | 8 | 3 |
| Other unrelated causes | 22 | 3 | 3 | 8 | 8 |
| No final details obtained | 26 | 3 | 2 | 8 | 13 |
| Totals | 319 | 42 | 36 | 115 | 126 |

Council's Working Party on Leukaemia in Adults [1980*b*]), none of the groups benefitted from the addition of vincristine. Overall, after adjustment for prognostic group, no significant difference between treatments was observed ($\chi^2=0.17$, $P>0.5$ after adjustment for prognostic groups). (Table I).

Causes of death were analysed in relation to first line treatment allocation (Table II). The slightly poorer survival in patients receiving vincristine is not attributable to an excess of deaths in that group of patients dying from causes other than uncontrolled myelomatosis.

### *Effect on duration of survival of extending first line treatment for a further year after patients had reached plateau*

Patients whose disease did not progress during first line treatment and who did not die from other causes were treated until their serum paraprotein and urinary free light chain output reached stable levels. A total of 268 patients reached stable plateau phase, as defined in the methods section. Of these 226 patients were re-randomised either to continue first line treatment for a further year or to stop treatment. In either case patients were followed up regularly and restarted on treatment or changed to second line treatment if disease progression occurred. The survival after second randomisation for patients in the stop and continue groups is shown in Figure 2. There was a small trend toward a better survival in the "stop" group but this was not significant ($\chi^2=1.47$, $P=0.2$). This result was not affected by the treatment allocated at initial presentation ($\chi^2=1.53$, $P=0.2$ after adjustment for initial therapy). The level of response to treatment achieved in patients who were rerandomised at plateau was assessed (Table III). Twenty-nine percent of the patients reaching plateau achieved complete serological remission. There is a trend to better survival in patients achieving good responses to chemotherapy. In each of the subgroups defined by level of response the stop group patients fared slightly better than those who were randomised to continue therapy. However, follow-up of the stop: continue randomisation is too short to draw final conclusions.

**Figure 2** Survival from second randomisation according to maintenance policy. Stop cytotoxic therapy until signs of progression (——) or continue cytotoxic therapy for one further year (······). Numbers in parentheses indicate number of patients randomised to each arm. $\chi^2=1.47$; $P=0.2$.

**Table III** Level of serological response at plateau in patients randomised to stop or continue treatment

| Second randomisation | *No response but stable disease* | *Partial response without total loss of urine light chain or serum paraprotein* | *Partial response with total loss of urine light chain or serum paraprotein* | *Complete response with total loss of both urine light chain and serum paraprotein* |
|---|---|---|---|---|
| STOP | 7 | 36 (712) | 38 (769) | 29 (957) |
| | (571) | | | |
| CONTINUE | 12 | 39 (604) | 23 (766) | 33 (731) |

Figures shown in the table are: numbers of patients in each group (60% actuarial survival in days post second randomisation).

No response = Urinary light chain output $g^{-1}$ creatinine $<150\%$ $>50\%$ of presentation values and serum paraprotein $<125\%$ $>75\%$ presentation values.

Partial response = Urinary light chain output $g^{-1}$ creatinine $\leqslant 50\%$ of presentation values but $\geqslant 0.04\,g\,g^{-1}$ creatinine. Serum paraprotein $<75\%$ starting values but still detectable.

Complete response = Urine light chain $<0.04\,g\,g^{-1}$ creatinine and no detectable serum paraprotein.

In addition to the patients shown in the Table, 7 patients with non secretory myelomatosis were also rerandomised: 3 to the stop group 4 to the continue group. Serum paraproteins were not assessed in 2 further patients at presentation.

The distribution of presentation serum paraprotein types and urinary light chain output in patients in this trial have been reported previously (Cooper *et al.*, 1984).

### Discussion

An analysis of a number of 4 and 5-drug regimens used by the South West Oncology Group (Alexanian *et al.*, 1977; Alexanian & Dreicer, 1984) suggested that patients on regimens including vincristine fared better than those treated with protocols not including this agent. This viewpoint was expressed in a BMJ editorial (1978) and the results reported by Lee *et al.* (1974) Salmon (1975) and later Case *et al.* (1977) were also cited as confirmatory evidence. The Cancer and Leukaemia group B on the other hand carried out a randomised controlled trial in which vincristine and prednisone were added late during first line therapy. They failed to show that the addition of these agents at 22 weeks had either increased the rate of subsequent objective responses or prolonged survival in patients treated with melphalan or nitrosoureas (Cornwell *et al*, 1982). Our trial was designed to assess the value of the addition of vincristine, at doses and intervals used by the South West Oncology group to standard treatment with intermittent melphalan and prednisone and no benefit could be found.

Recently Barlogie *et al.* (1984) have reported effective treatment of advanced myeloma refractory to alkylating agents, with a regimen consisting of high dose dexamethasone, and prolonged infusion of vincristine and doxorubicin. It is difficult to assess what role vincristine may have had in achieving these responses.

There is no clear concensus in the literature about the optimal duration of first line treatment in myelomatosis. To some extent this will depend upon the treatment used. As response rates vary from patient to patient, it seems logical to relate length of first line treatment to plateau phase rather than to a fixed time from starting therapy. In the third MRC trial patients were treated initially for 1 year only and were then rerandomised to receive maintenance or no further chemotherapy (MRC Working Party on Leukaemia in Adults, 1980*a*). The duration of survival was slightly better in those patients not receiving maintenance but this difference is not significant. The South West Oncology Group (1975) assessed the value of continuing melphalan and prednisone for more than 1 year and concluded that this was of no major value.

Other reports have appeared supporting the policy of limiting the duration of first line treatment (Alexanian *et al.* 1978; Paccagnella *et al.* 1983). Arguments in favour of restricting the length of first line treatment include: (i) an improved chance of achieving second responses to chemotherapy when disease subsequently progresses; (ii) reduction in myelotoxicity, infection and secondary leukaemia and (iii) improved quality of life in patients on stable plateau phase who are not receiving chemotherapy. The present study has provided objective evidence from a large randomised trial that first line therapy with intermittent melphalan and prednisone should not be continued after plateau phase has been reached. It remains to be shown whether the introduction of different cytotoxic agents at this stage might be of benefit. However, cytokinetic studies of patients' disease at plateau phase indicate that residual disease may be inherently resistant to further chemotherapy (Hokanson *et al.*, 1977; Durie *et al.*, 1980).

The authors wish to thank the physicians throughout the United Kingdom who entered patients to this study and the members of the department of Immunology at the University of Birmingham who carried out serial paraproteins and urinary free light chain estimations on all patients in the trial.

### References

ALEXANIAN, R., SALMON, S., BONNET, J., GEHAN, E., HAUT, A. & WEICK, J. (1977). Combination chemotherapy for multiple myeloma. *Cancer*, **40**, 2756.

ALEXANIAN, R., GEHAN, E., HAUT, A., SAIKI, J. & WEICK, J. for the Southwest Oncology Group. (1978). Unmaintained remissions in multiple myeloma. *Blood*, **51**, 1005.

ALEXANIAN, R. & DREICER, R. (1984). Chemotherapy for myeloma. *Cancer*, **53**, 583.

BARLOGIE, B., SMITH, L. & ALEXANIAN, R. (1984). Effective treatment of advanced multiple myeloma refractory to alkylating agents. *N. Engl. J. Med.*, **31**, 1353.

EDITORIAL. (1978). Progress in myeloma at last. *Br. Med. J.*, **i**, 1653.

BONNET, J.D., ALEXANIAN, R., SALMON, S.E., HANT, A. & DIXON, D.O. (1984). Addition of Cisplatin and Bleomycin to vincristine-carmustine-Doxorubicin-prednisone (VBAP) combination in the treatment of relapsing or resistant multiple myeloma: A South West Oncology group study. *Cancer Treat. Rep.* **68**, 481.

CASE, D.C., LEE, B.J. & CLARKSON, B.D. (1977). Improved survival times in multiple myeloma treated with melphalan, prednisone, Cyclophosphamide, vincristine and BCNU: M-2 protocol. *Am. J. Med.*, **63**, 897.

COOPER, E.H., FORBES, M.A., CROCKSON, R.A. & MACLENNAN, I.C.M. (1984). Proximal renal tubular function in myelomatosis: Observations in the fourth Medical Research Council trial. *J. Clin. Pathol.*, **37**, 852.

CORNWELL, III, G.G., PAJAK, T., KOCHIVA, S. & 10 others. (1982). Comparison of oral melphalan CCNU and BCNU with and without vincristine and prednisone in the treatment of multiple myeloma. *Cancer*, **50**, 1669.

DURIE, B.G.M., RUSSELL, D.H. & SALMON, S.E. (1980). Reappraisal of plateau phase in myeloma. *Lancet*, **ii**, 65.

HOKANSON, J.A., BROWN, B.W., THOMPSON, J.R., DREWINKO, B. & ALEXANIAN, R. (1977). Tumor growth patterns in multiple myeloma. *Cancer*, **39**, 1077.

LEE, B.J., SAHAKIAN, G., CLARKSON, B.D. & KAROFF, I.H. (1974). Combination chemotherapy of multiple myeloma with alkeran, cytoxan, vincristine, prednisone and BCNU. *Cancer*, **33**, 533.

MEDICAL RESEARCH COUNCIL'S WORKING PARTY ON LEUKAEMIA IN ADULTS. (1980*a*). Treatment comparisons in the third MRC myelomatosis trial. *Br. J. Cancer*, **42**, 823.

MEDICAL RESEARCH COUNCIL. (1980*b*). Prognostic features in the third MRC myelomatosis trial. *Br. J. Cancer*, **42**, 831.

MEDICAL RESEARCH COUNCIL'S WORKING PARTY ON LEUKAEMIA IN ADULTS. (1984). Analysis and management of renal failure in the fourth MRC myelomatosis trial. *Br. Med. J.*, **288**, 1411.

PACCAGNELLA, A., CARTEI, G., FOSSER, V. & 4 others. (1983). Treatment of multiple myeloma with m-2 protocol and without maintenance therapy. *Eur. J. Cancer Clin. Oncol.*, **19**, 1345.

SALMON, S.E. (1975). Expansion of growth fraction in multiple myeloma with alkylating agents. *Blood*, **45**, 119.

SOUTHWEST ONCOLOGY GROUP STUDY. (1975). Remission maintenance therapy for multiple myeloma. *Arch. Intern. Med.*, **135**, 147.